# Whole-Cell Photobleaching Reveals Time-Dependent Compartmentalization of Soluble Proteins by the Axon Initial Segment

**DOI:** 10.3389/fncel.2020.00180

**Published:** 2020-07-10

**Authors:** LaShae Nicholson, Nicolas Gervasi, Thibault Falières, Adrien Leroy, Dorian Miremont, Diana Zala, Cyril Hanus

**Affiliations:** ^1^Department of Neurology, Yale University School of Medicine, New Haven, CT, United States; ^2^Center for Interdisciplinary Research in Biology, College de France, Inserm U1050, CNRS UMR 7241, Labex Memolife, Paris, France; ^3^Institute for Psychiatry and Neurosciences of Paris, Inserm UMR 1266, University of Paris, 4 GHU PARIS Psychiatrie & Neurosciences, Paris, France

**Keywords:** neuronal polarity, protein compartmentalization, axon initial segment, correlative imaging, super-resolution, fluorescence loss in photobleaching, computational modeling

## Abstract

By limiting protein exchange between the soma and the axon, the axon initial segment (AIS) enables the segregation of specific proteins and hence the differentiation of the somatodendritic compartment and the axonal compartment. Electron microscopy and super-resolution fluorescence imaging have provided important insights in the ultrastructure of the AIS. Yet, the full extent of its filtering properties is not fully delineated. In particular, it is unclear whether and how the AIS opposes the free exchange of soluble proteins. Here we describe a robust framework to combine whole-cell photobleaching and retrospective high-resolution imaging in developing neurons. With this assay, we found that cytoplasmic soluble proteins that are not excluded from the axon upon expression over tens of hours exhibit a strong mobility reduction at the AIS – i.e., are indeed compartmentalized – when monitored over tens of minutes. This form of compartmentalization is developmentally regulated, requires intact F-actin and may be correlated with the composition and ultrastructure of the submembranous spectrin cytoskeleton. Using computational modeling, we provide evidence that both neuronal morphology and the AIS regulate this compartmentalization but act on distinct time scales, with the AIS having a more pronounced effect on fast exchanges. Our results thus suggest that the rate of protein accumulation in the soma may impact to what extent and over which timescales freely moving molecules can be segregated from the axon. This in turn has important implications for our understanding of compartment-specific signaling in neurons.

## Introduction

Located a few tens of microns from the soma, the axon initial segment (AIS) is the specialized domain of the neuronal membrane where axon potentials are generated. The AIS is also a physical barrier that opposes the free exchange of proteins and membranes between the soma and the axon (Zhang and Rasband, 2016; Leterrier et al., 2017). The AIS hence enables the segregation of specific cellular components (e.g., pre and postsynaptic proteins) (Hedstrom et al., 2008; Sobotzik et al., 2009) and materializes the boundary between the somatodendritic compartment and the axonal compartment.

Consistent with its crucial role in separating and maintaining these compartments, the formation of the AIS is coordinated with the specification of the axon and occurs early on during neuronal development (Petersen et al., 2014), before extensive dendritic growth and synaptogenesis (Dotti et al., 1988).

The AIS is composed of transmembrane proteins, including neurofascin (a cell-adhesion molecule) and sodium and potassium voltage-gated channels, which are connected to the actin cytoskeleton via βIV spectrin and Ankyrin G (AnkG) – the core scaffolding protein of the AIS (Jenkins and Bennett, 2001; Hedstrom et al., 2007; 2008). As shown by superresolution microscopy studies, the ultrastructure of the AIS consists of repeated actin rings, whose 190 nm periodicity is determined by elongated head-to-head tetramers of βIV-spectrin (Xu et al., 2013; Leterrier et al., 2015).

By virtue of its tight interaction with surface transmembrane proteins, this submembranous cytoskeleton generates periodic diffusion barriers in the plasma membrane (Albrecht et al., 2016), which oppose the passage of surface proteins through the AIS (Winckler et al., 1999; Nakada et al., 2003). The AIS also controls the exchange of intracellular membranes between the soma to the axon. Likely through the interaction of AnkG with microtubules and microtubule regulatory proteins (Jenkins and Bennett, 2001; Leterrier et al., 2011), the AIS prevents the active transport of specific membrane cargo to the axon. This selectivity is determined by the specific kinesins membranes interact with. For example, while KIF5 cargo is allowed to enter the axon, KIF17 interacting membranes are prevented from passing through the AIS (Song et al., 2009).

Although the underlying mechanisms are not yet clear, the AIS may also prevent soluble cytoplasmic molecules from entering the axon. For example, 10 kDa dextran – a branched polysaccharide – injected in the soma passes the AIS and rapidly equilibrates throughout the entire neuron. In contrast, 70 kDa dextran diffuses throughout the somatodendritic compartment but is excluded from the axon (Song et al., 2009; Sun et al., 2014). The case of soluble cytoplasmic proteins is not well understood. Photobleaching studies have reported that the apparent mobility of green fluorescent protein (GFP) may be reduced in the proximal axonal segment of DIV 5 but not DIV 3 neurons, mirroring the formation of the AIS between these two time points (Song et al., 2009). However, this reduced mobility was observed only in a subset of DIV 5 neurons and was accompanied by a global reduction of protein dynamics throughout the axon (Song et al., 2009). It is thus unclear at present to what extent this general reduction in GFP dynamics reflects filtering at the AIS.

Over the past years, superresolution imaging enabled significant progress in our understanding of the ultrastructure of the AIS (Leterrier et al., 2017; Rasband, 2019). However, how this ultrastructure impacts protein dynamics is much less known. There is thus a need for standardized live/structural correlative imaging assays for comparison across studies. Here we describe a reliable and accessible approach to do so. Using this assay, we found that small (27 kDa) cytoplasmic proteins are indeed filtered at the AIS. Interestingly, while these proteins distribute throughout the entire neuron when expressed over tens of hours, their passing through the AIS is rate limiting and opposes their exchange over periods of tens of minutes. In other words, while a given soluble protein may be stable enough to ultimately enter the axon, shorter-lived pools of proteins of similar shape and size may be effectively compartmentalized by the AIS. Using computational modeling, we provide evidence that both the AIS and neuronal morphology regulate protein exchanges between the soma and the axon but do so at different time scales, with the AIS having a more pronounced effect on fast exchanges. Consistent with previous reports (Song et al., 2009; Sun et al., 2014), we found that protein filtering at the AIS is developmentally regulated and requires an intact F-actin cytoskeleton. We provide proof of principle that our FLIP assay is well suited for retrospective super-resolution imaging and, based on a case study, discuss how the composition and ultrastructure of the cortical spectrin/actin cytoskeleton may have a general role in regulating the diffusion of cytoplasmic proteins in developing neurons.

## Results

### Probe Selection and Practical Considerations on the Use of Photobleaching to Study the AIS

Expanding our previous work on the neuronal endoplasmic reticulum (Cui-Wang et al., 2012), we devised photobleaching assays to study protein compartmentalization in days-*in vitro* (DIV) 3–5 hippocampal neurons.

**FIGURE 1:**
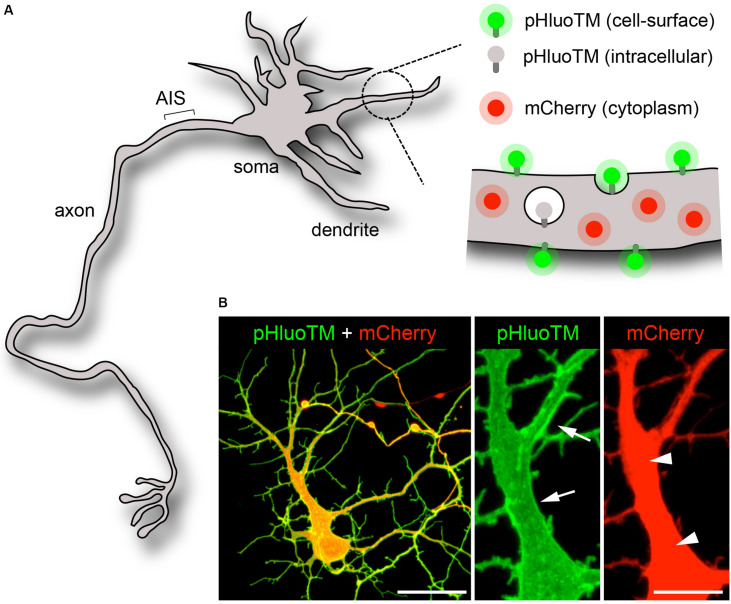
Visualization of the neuronal cytoplasm and plasma membrane. **(A,B)** Scheme **(A)** and confocal pictures (**B**, maximal projections) of an immature neuron (DIV5) expressing pHluoTM (green) and mCherry (red). PHluoTM – a 39 KDa type I transmembrane protein derived from superecliptic (pH-sensitive) GFP – selectively fluoresces and marks the plasma membrane (arrows). mCherry, a 27KDa soluble protein, distributes throughout the cytoplasm (arrowheads). Scale bar 50 or 10 μm.

To assess the dynamics of a small cytoplasmic protein, we monitored mCherry or tdTomato (tdTom), 27 and 55 kDa red fluorescent cytoplasmic proteins, respectively (Figure 1; Shaner et al., 2004; Cui-Wang et al., 2012). For direct comparison, mCherry or tdTom were imaged together with pHluoTM (Cui-Wang et al., 2012), a 39 KDa transmembrane protein derived from pH-sensitive GFP (Miesenböck et al., 1998), which selectively fluoresces at the neutral pH of the extracellular milieu (Figure 1A). Consistent with previous studies (Cui-Wang et al., 2012), pHluoTM was efficiently expressed at and marked the cell surface (Figure 1B).

Fluorescence recovery after photobleaching (FRAP) (Poo and Cone, 1974; Axelrod et al., 1976) still represents a gold standard to study the local dynamics of candidate proteins in homogenous cellular structures *in situ.* However, this technique is less suited for assessing protein compartmentalization at the scale of the entire axonal tree or the entire neuron. Notably, generic FRAP experiments focus on a small region of the cell – typically a few microns wide – and thus critically depends on the positioning of this region (Cui-Wang et al., 2012; Lorén et al., 2015). This is particularly problematic in the case of the AIS, whose length and position relative to the soma changes over time and can vary greatly from one neuron to another (Grubb and Burrone, 2010; Yoshimura and Rasband, 2014; Dumitrescu et al., 2016). One possible solution is to image a fluorescent marker of the AIS together with the probes that are monitored (Dumitrescu et al., 2016), consequently reducing the number of fluorescent channels available for the experiment.

**FIGURE 2:**
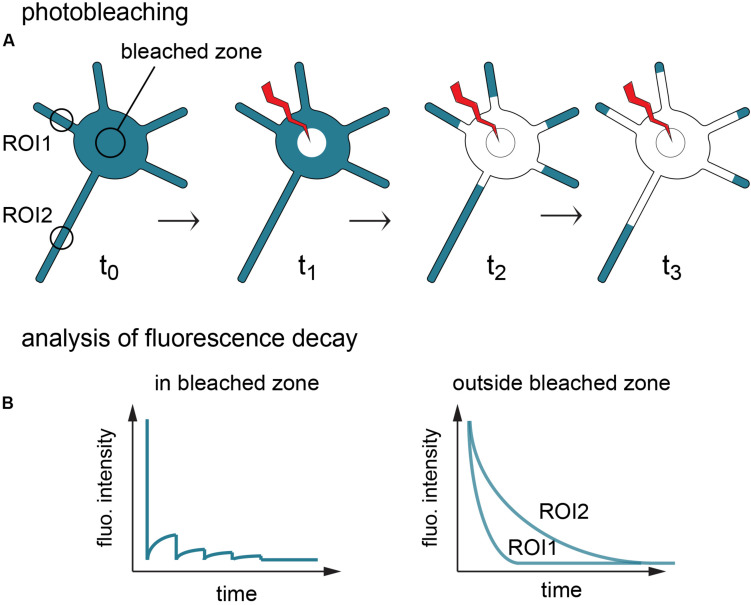
Principle of fluorescence loss in photobleaching (FLIP). **(A)** Fluorescent proteins of interest (turquoise) are repetitively photobleached in the soma, creating a sink that progressively depletes the cell fluorescence. **(B)** Fluorescence decay in the bleached zone is determined by local photobleaching and protein mobility. Fluorescence decay in the rest of the cell (here shown in 2 regions of interest) is determined solely by molecule exchanges with the bleached zone, thus materializing constraints on protein dynamics throughout the cell.

To avoid these shortcomings, we used fluorescence loss in photobleaching (FLIP) (Cui-Wang et al., 2012). In this assay a small region of the cell, here in the soma, is repetitively photobleached, progressively depleting the fluorescence of a given probe throughout the entire cell by virtue of exchanges between the bleached area and unbleached neighboring structures (Figure 2A). The resulting fluorescence decay in the bleached zone reflects both local photobleaching and the mobility of the probes, while decay in other regions solely reflects the speed and extent of protein exchange with the bleached zone (Figure 2). In contrast to FRAP, FLIP thus provides information on protein dynamics at a local scale and at the scale of the entire cell (i.e., in the bleached zone vs. the rest of the cell), revealing diffusion barriers and bottlenecks affecting these dynamics (Wüstner et al., 2012).

### FLIP Reveals Contrasted Constraints on the Compartmentalization of Soluble Cytoplasmic vs. Transmembrane Proteins

We thus used FLIP to compare the global exchanges of pHluoTM and mCherry in DIV5 neurons that were bleached every 30 s in a 7.5 μm wide circle placed on the soma together with acquisition of z-stacks of mCherry and pHluoTM (see section “Materials and Methods”). The time needed for imaging and photobleaching at each time point was much shorter (typically a few seconds) than the 30 s cycles used for time-lapse acquisition, enabling us to perform experiments in up to 3–5 cells in parallel.

**FIGURE 3:**
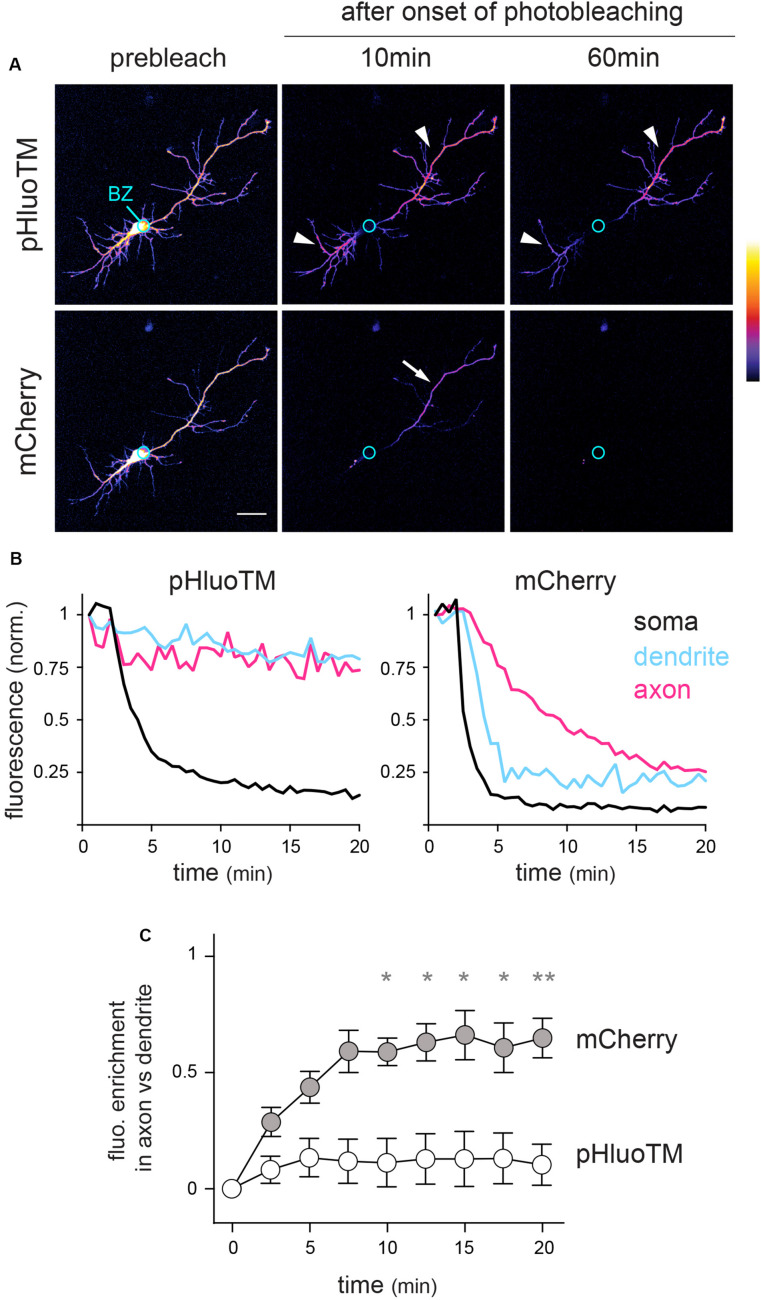
FLIP reveals contrasted constraints on the dynamics of cytoplasmic and surface transmembrane proteins. **(A)** mCherry and pHluoTM fluorescence (maximum intensity projections – color coded) in a DIV5 neuron before and 10 and 60 min after onset of photobleaching in the soma (bleached zone, BZ, marked by a circle). Scale bar 50 μm. **(B)** Example plots of mCherry and pHluoTM fluorescence decay in the bleached zone and 75 μm away from the soma in a presumed dendrite and in the presumed axon, same cell as in **A**). **(C)** Population analysis: fluorescent enrichment in axons vs. dendrites ([F_axon_ - F_dendrite_]/[F_axon_ + F_dendrite_]; mean ± SEM, N = 8 cells) at the indicated time after onset of photobleaching in intermediate segments of dendrites and axons (60 or 100 μm away from the soma). *p < 0.05; **p < 0.01; Kruskal–Wallis’ multicomparison test). In **(A–C)**, note that pHluoTM fluorescence is rapidly and completely depleted from the soma and proximal neurites but remains largely unaffected in distal neurites (arrowheads). In contrast, mCherry fluorescence is rapidly depleted from the entire neuron, albeit with much slower kinetics in a single neurite (arrow): the presumed axon.

Based on their relative diffusion coefficients (5–15 vs. 0.02–0.5 μm^2^/s for an average protein in the cytoplasm or in the extrasynaptic plasma membrane, respectively) (Moran et al., 2010; Choquet and Triller, 2013) and specific dynamics (diffusion in the cytoplasm vs. diffusion in the plane of the plasma membrane), we expected that photobleaching would have contrasted effects on these two proteins. Consistently, while pHluoTM fluorescence was depleted in and around the cell body but could still be detected in distal neurites 60 min after the onset of photobleaching (t_0_ + 60 min, Figures 3A–C and Supplementary Movie S1), mCherry fluorescence was rapidly lost over the entire neuron, albeit with a slower decay in a single neurite, the longest one: the presumed axon (see thereafter) (Figures 3B,C).

FLIP thus reveals the extent and spatial length over which the somatic pool of a given protein can be selectively affected without impacting neurites, hence objectifying the effective compartmentalization of this protein.

These data show that FLIP efficiently reveals how the specific biophysical constraints on the mobility of given proteins – here a transmembrane protein and a cytoplasmic protein – determine the effective compartmentalization of these proteins throughout the entire neuron. Our results show that, over the time interval and with the rate of photobleaching considered here, the diffusion of a transmembrane protein is slow enough that spatially distant pools (e.g., soma vs. distal neurites) can be modified selectively. In contrast, the diffusion of a small cytoplasmic protein is so fast that its compartmentalization in the soma or a given neurite requires diffusion barriers.

### Pixel-by-Pixel Analysis of Fluorescent Decay Reveals the Presence and Location of Diffusion Barriers Opposing the Diffusion of Cytoplasmic Proteins Throughout the Entire Neuron

**FIGURE 4:**
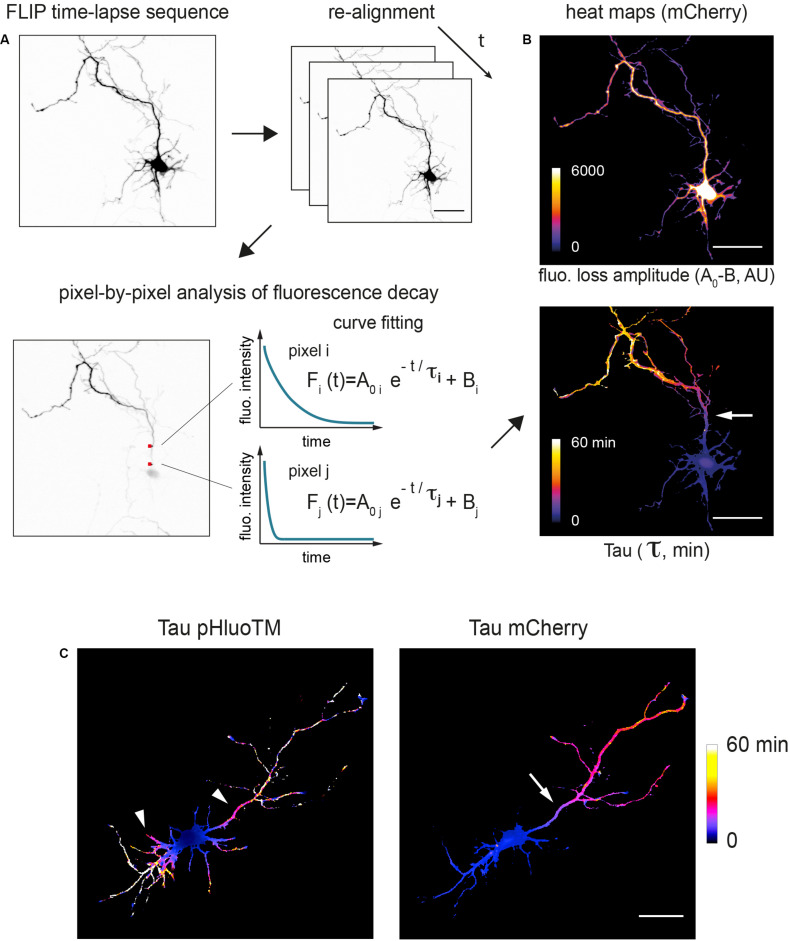
Pixel-by-pixel analysis of fluorescence decay shows localized filtering of cytoplasmic proteins at the AIS. **(A)** Image processing workflow. Maximum projections of z-stacks acquired at each time points are aligned using the pHluoTM channel, filtered with Gaussian functions and fluoresence decay at each pixel fitted with a mono-exponential function. Estimated decay tau and amplitude (color-coded) are then displayed as heat maps delineating the cell silhouette. **(B)** Shown are example maps obtained with mCherry in a DIV3 neuron. Scale bar 50 μm. In the lower panel (tau map) note the localized slowing down of mCherry (increased tau values) at the location of the presumed AIS (arrow). **(C)** Other examples of tau maps (color-coded, time in min) obtained for pHluoTM and mCherry at low and higher magnifications (same cell as in Figure 3A). Scale bar 50 μm. In the left hand panel (pHluoTM), note the symmetrical slowing down of pHluoTM decay in dendrites and the axon (arrowheads). In the right hand panel, note the asymmetrical slowing down of mCherry decay, markedly slower in the axon, and, as in **(B)**, the slowing down of mCherry marking the location of presumed AIS (arrow).

**FIGURE 5:**
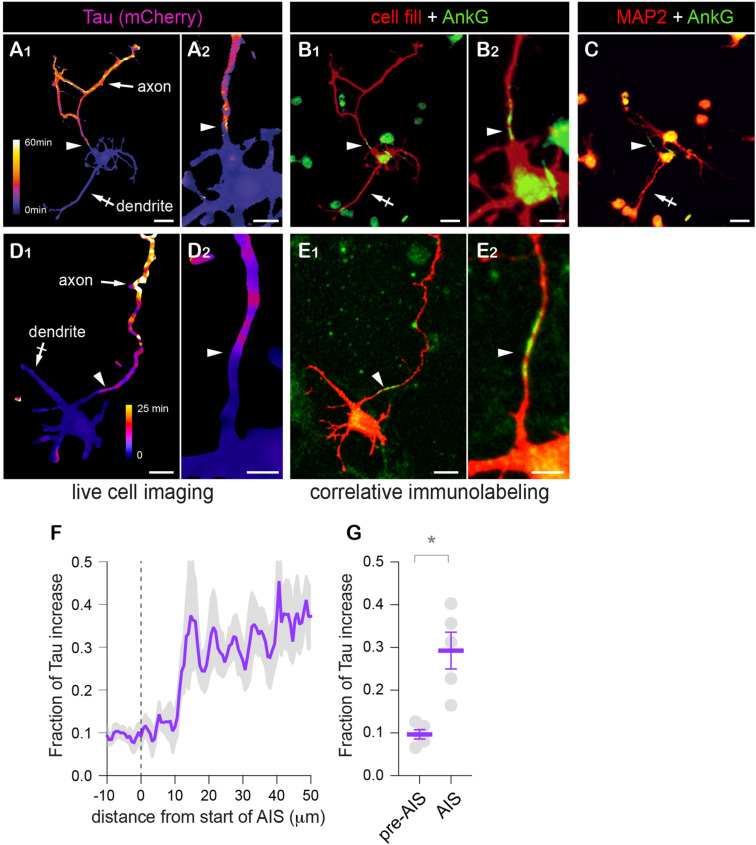
Retrospective immuno-labeling confirms the identity of the presumed AIS. **(A–E)** Variation of mCherry **(A1,A2)** or tdTom **(D1,D2)** decay speed (tau heat maps) and distributions of mCherry (prebleach fluorescence, red in **B1,B2**) or co-expressed cell fill (GFP, red in **E1,E2**), Ankyrin G (AnkG, green in **B1,B2,E1,E2**) and MAP2 (red in **C**) in DIV3 **(A–C)** or DIV 5 **(E,D)** neurons at low **(A1,B1,D1,E1,C)** or higher magnification **(A2,B2,D2,E2)**. Scale bar 25 or 12.5 μm. **(F)** Fractional increase of Tau along the axon in relation to the start of the AIS (as in **B,E**). **(G)** Averaged fractional increase before (−10 to 0 μm) and in the AIS (−10 to 20 μm). Shown are means ± SEM from *N* = 7 neurons. *p < 0.05; Wilcoxon’s matched-paired test. In **(A–E)**, note that the presumed axon identified by the slower decay of mCherry or tdTom (arrow in **A1,D1**) does not express MAP2 (in contrast to a presumed dendrite, crossed arrow in **A1,C**) and displays an ankyrin G-positive AIS (arrowheads in **B1,B2,E1,E2**), and can thus be identified as the axon. Tau increased is particularly marked a few tens of microns from the soma and is coordinated to the location of the AIS identified by AnkG (arrowheads). In **(F)**, note the ∼10 μm offset between the start of the AIS and Tau increase. In **(G)**, note the higher values of Tau after the first 10 μm of the AIS.

**FIGURE 6:**
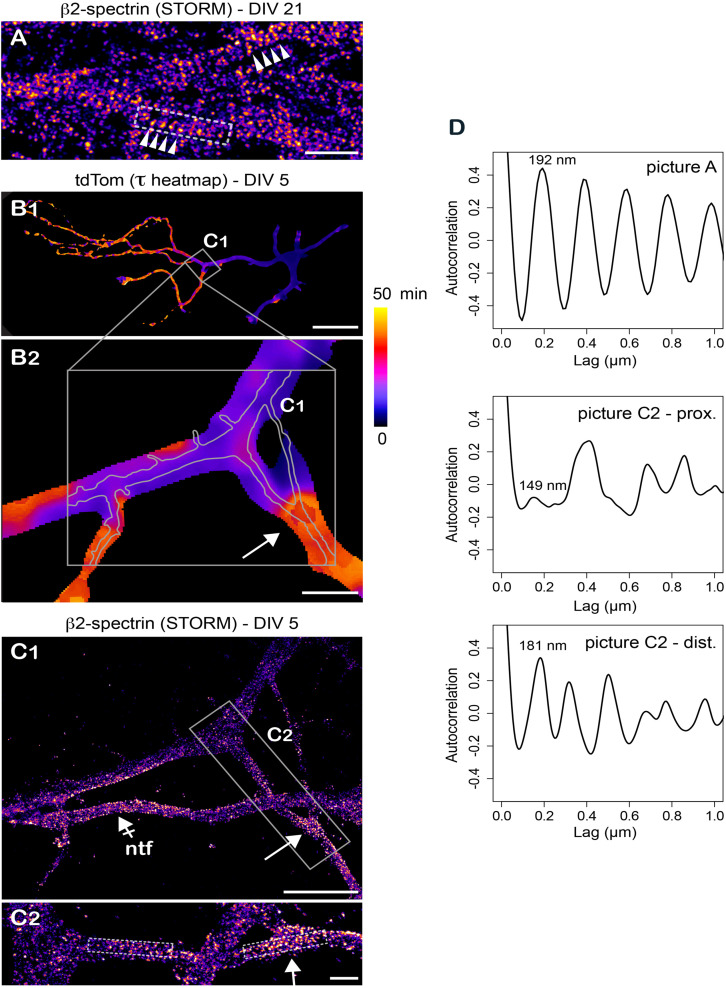
Retrospective stochastic optical reconstruction microscopy demonstrates the feasibility of correlative FLIP/super-resolution imaging. **(A)** Stochastic optical reconstruction microscopy (STORM) of βII-spectrin in axons of DIV 29 hipocampal neurons showing the repetitive organization of the subcortical cytoskeleton (spectin ring spacers, arrowheads). **(B)** Variation of tdTom diffusion throughout a DIV 5 neuron (tau heat maps) at low **(B1)** and higher **(B2)** magnification. Shown in gray in B2 are the position and contours of the axon imaged at higher resolution in **(C)**. **(C)** Retrospective STORM imaging of βII-spectrin in the area highlighted in **(B)** at low **(C1)** and higher magnification **(C2)**. Shown by a crossed-arrow in **(C1)** is a non-transfected neuron (ntf). In **(B,C1,C2)**, note the presence of repetitive βII-spectrin structures in zones of transition from fast to slower diffusion of tdTom (arrows). **(D)** Auto-correlation signal along the longitudinal axes of areas highlighted in gray in **(A,C2)**. For **(C2)**, note the stronger periodicity of the signal in the distal dendritic segment and the strong resonance at 181nm in this segment. Scale bars 1, 50, 10, 5, and 1 μm in **(A,B1,B2,C1,C2)**, respectively.

To directly visualize and quantify how protein dynamics spatially vary throughout the entire neuron, fluorescence decay was measured pixel by pixel. In a few words, maximal projections of z-stacks were aligned and fluorescence decay at each pixel fitted by a mono exponential decay curve (Figure 4, see section “Materials and Methods” and Supplementary Figure S1; Wüstner et al., 2012). This enabled us to generate cellular heat maps to quantitatively assess various aspects of fluorescence decay, in particular the decay time constant t (tau) (Figure 4). As shown in Figures 4–6, tau heat maps provided a direct visualization of the various trends described above: pHluoTM exhibited a progressive drop of decay rates that was relatively homogenously distributed around the bleached zone, i.e., similar in all neurites (Figure 4C, left). In contrast, mCherry decay was homogenous throughout the neuron at the exception of the presumed axon (Figures 4B,C).

Remarkably, the decay tau heat maps for mCherry showed that the reduction of the protein exchanges between the somatodendritic compartment and the axon was more pronounced at a specific location of the axon (Figures 4B,C). The occurrence of this filter in a single neurite and its distance from the soma (∼30 μm) (Grubb and Burrone, 2010) clearly hinted that it corresponded to the AIS. To determine whether this was indeed the case, neurons were fixed after live-cell imaging and immunoreacted for MAP2 (a marker or the somatodendritic compartment) and AnkG (an AIS protein) to identify neurites as dendrites or axons and to determine the location of the AIS. As shown in Figure 5, the presumed axon in these neurons was MAP2 negative as expected and the sharp increase in tau matched the location of the AIS defined by the accumulation of AnkG (Figures 5A–E). Intriguingly, Tau increase was offset by ∼10 microns from the start of the AIS. The reason for this offset is unclear at present. As shown for surface membrane proteins, the subcortical actin/spectrin cytoskeleton forms repetitive structures (see hereafter) individually opposing diffusion along the long axis of the axon (Albrecht et al., 2016). It is thus possible that a similar cumulative effect exists also for cytoplasmic proteins and may only be measurable after a certain distance within the AIS.

Thus, our FLIP approach enables quantitative access to protein filtering at the AIS, without positioning the bleached zone at this specific location, providing an unbiased approach to study protein dynamics throughout the entire neuron.

Over the past years, super-resolution imaging revealed multiple and previously unrecognized features of the neuronal cytoskeleton ultrastructure – notably the existence of subcortical actin/spectrin rings (Xu et al., 2013; Leterrier et al., 2015; He et al., 2016). It is thus essential to now develop functional assays to determine how these structural features impact protein dynamics. As proof of principle that our FLIP assay is well adapted for such approaches, we performed correlative whole cell FLIP/super-resolution imaging using stochastical optical reconstruction microscopy (STORM) after labeling of βII spectrin (see section “Materials and Methods” for details). While βIV-spectrin periodic rings are located at the AIS, those formed by βII-spectrin are found in some segments of both dendrites and axons (He et al., 2016). We thus focused on the later because of their widespread distribution. In our experiments, periodic rings were easily observed in distal axonal segments of DIV 29 neurons (Figure 6A). Consistent with other reports (Xu et al., 2013; Leterrier et al., 2015; He et al., 2016), these structures were less pronounced in DIV 5 neurons (Figures 6C,D and Supplementary Movie S3). As shown in an axonal segment where we could clearly visualize these variations, we found that transition from faster to slower diffusion of cytoplasmic proteins seemed correlated with transition from a weaker to a stronger periodicity of βII-spectrin labeling (Figures 6B–D). While more work will be required to investigate such correlations and their molecular underpinning, our results show that our FLIP assay is particularly well adapted for retrospective immuno-labeling and super-resolution imaging, opening new avenues for correlative microcopy. In particular, it will be interesting to study protein diffusion in relation to the ultrastructure of submembranous elements formed by other spectrin isoforms at multiple stages of the maturation of actin/spectrin rings during neuronal development.

### The Compartmentalization of Cytoplasmic Proteins in the Somatodendritic Compartment Is Developmentally Regulated and Is Controlled by F-Actin

**FIGURE 7:**
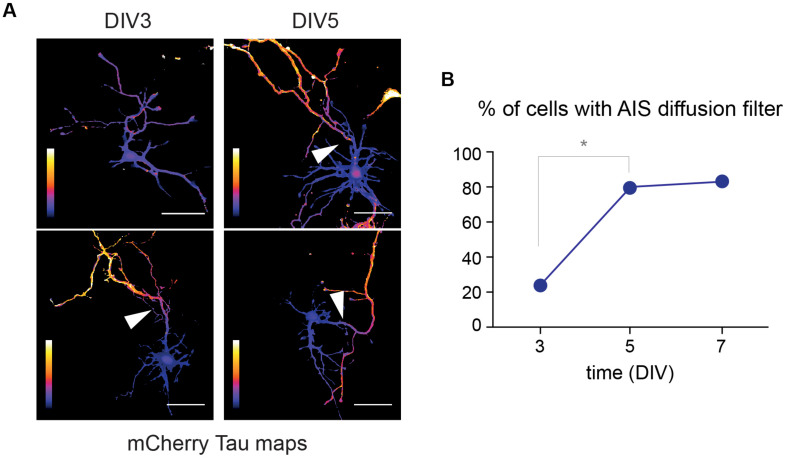
The filtering of cytoplasmic protein in the axon is developmentally regulated. **(A)** Tau heat maps of mCherry decay in DIV3 and DIV5 neurons illustrating the occurrence or lack of occurrence of a cytoplasmic diffusion barrier (arrowheads). Scale bar 50 μm. **(B)** Proportion of neurons exhibiting such a diffusion barrier at DIV3, 5, and 7, documenting the progressive maturation of the AIS (*p < 0.01; Fisher’s exact test, *N* = 13, 15, and 6 neurons, at DIV3, 5, and 7, respectively).

In cultured hippocampal neurons and as shown for surface proteins and trafficking vesicles, the AIS starts forming at DIV3 and is fully functional at DIV5 (Song et al., 2009; Leterrier and Dargent, 2014). To determine whether this maturation also impacts the filtering of cytoplasmic proteins, we performed FLIP experiments in DIV3, DIV5, and DIV7 neurons as described above, and compared the proportion of neurons exhibiting a diffusion filter at the AIS. We found that cytoplasmic proteins were filtered at the AIS in 23, 80, and 83% of DIV 3, 5, and 7 neurons, respectively (Figures 7A,B). This thus indicates that the compartmentalization of cytoplasmic proteins between the soma and the axon is developmentally regulated and mirrors the morphological formation and maturation of the AIS.

**FIGURE 8:**
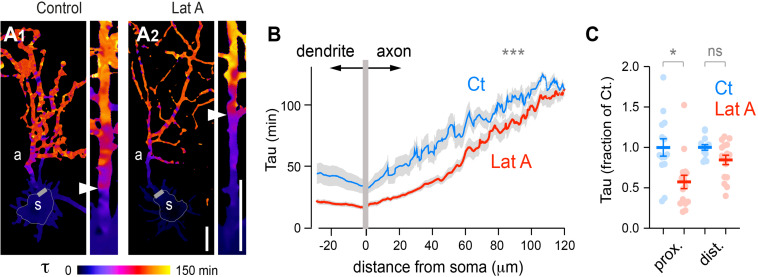
Disruption of F-actin accelerates the diffusion of cytoplasmic proteins throughout the entire neuron and is more pronounced in proximal than in distal axonal segments. **(A)** Tau heat maps of tdTom decay in DIV5 neurons treated with vehicle (Control) or 7.5 μM Latrunculine A for 60–120 min (Lat A). Scale bar 25 μm. Shown are whole cell tau maps and straightened axons at higher magnification. Simplified shape of somata and start of presumed axons are indicated by thin and thicker gray lines, respectively. Arrowheads in straightened axons mark the transition from fast to slower diffusion. **(B)** Tau variations along the main dendrite and axon as a function of distance from the soma in neurons treated with vehicle (Ct) or LatA. Shown are means ± SEM from *N* = 13–16 neurons; ***p < 0.001; Kolmogorow’s test. Note the lower tau values in cells treated with LatA. **(C)** Average Tau (fraction of control) in proximal (20–25 μm) and distal (100–105 μm) axonal segments. Shown are means ± SEM. *N* = 13–16 neurons; **p < 0.01; Kruskal–Wallis, Dunn’s multi-comparison tests. Note the more pronounced effect of LatA on proximal segments.

Previous FRAP experiments directed on the presumed axon showed that protein filtering at the AIS requires an intact actin cytoskeleton (Song et al., 2009). Given the presence of F-actin throughout the entire neuron, it is not clear yet to which extent this effect is specific of the AIS. To answer this, we performed FLIP experiments in DIV 5 neurons expressing tdTomato (tdTom) after treatment with vehicle (DMSO: control or Ct) or 7.5 μM latrunculin A (LatA) for 1.5–2 h. As shown in Figure 8, tau heat maps showed that latrunculin accelerated tdTom diffusion throughout the entire neuron (Figures 8A,B), an effect that was probably not detectable by photobleaching only the axon (Song et al., 2009). Consistent with the aforementioned study, however, we found that the effect LatA effect in axons was more pronounced in proximal segments (Figure 8C). In contrast to Tau plots centered on AnkG labeling shown in Figure 5F, Tau increase along the axon of control cells appeared more progressive (Figure 8B), likely due to neuron-to-neuron variations in the exact position of the AIS (Grubb and Burrone, 2010; Yoshimura and Rasband, 2014; Dumitrescu et al., 2016).

These results thus show that our FLIP assay enables monitoring of acute variations in global and local protein dynamics at the scale of the entire neuron in an unbiased manner.

### The AIS and Neuronal Morphology Both Contribute to the Compartmentalization of Cytoplasmic Proteins, Albeit at Different Time Scales

**FIGURE 9:**
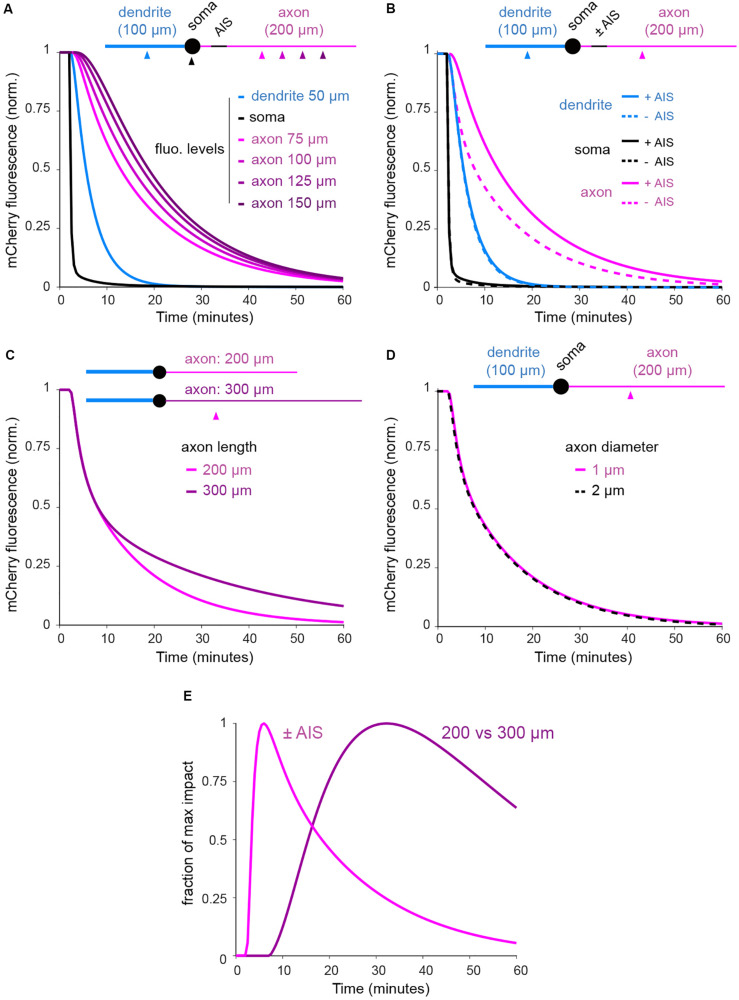
FLIP simulation in simplified neuronal shapes indicates that filtering at the AIS and axonal length shape the exchange of soluble axonal proteins at different temporal scales. **(A)** Normalized mCherry fluorescence in the soma and 75, 100, 125, and 150 μm from the soma in the dendrite and the axon of a neuron with AIS (see section “Materials and Methods” for details on diffusion-reaction computational model). **(B)** Decay in the soma and at 75 μm in the dendrite and the axon in a neuron with or without AIS. **(C)** Decay at 75 μm in 200 and 300 μm-long axons. **(D)** Decay at 75 μm in 1 and 2 μm-wide axons (the two plots are perfectly overlaid). **(E)** Normalized impacts of the AIS and axonal length (200 vs. 300 μm) as in **(B,C)**. In **(A)**, note the slowing down of protein exchanges in distal segments of the axon. In **(B,C)** note the slowing down of protein exchanges by the AIS and with increasing axonal length. In **(D)** note the absence of effect of axonal diameter. In **(E)**, note that the relative effects of the AIS and axonal length are shifted in time.

As shown in Figures 4–8, we found that the rate of protein exchange throughout developing neurons is locally reduced at the AIS but also progressively decreases along the axon. It is thus likely that geometrical constraints, and in particular axonal length, also influence the dynamics of cytoplasmic proteins. As a first step in addressing this, we used computational modeling to estimate the relative effects of the AIS vs. neuronal morphology. To do so, we developed a reaction-diffusion model where somata and neurites were modeled as spheres and cylinders, respectively, and molecule numbers in voxels and exchange between voxels set to represent mCherry diffusion throughout the cell and photobleaching in the soma (see section “Materials and Methods”). The model clearly reproduced trends observed in our experiments (Figure 9A) and confirmed that both axon length and filtering at the AIS regulate protein exchanges between the soma and the somatodendritic compartment (Figures 9B,C). At this level of analysis, we found that the diameter of the axon had no measurable impact on mCherry dynamics (Figure 9D). Interestingly, the relative effects of the AIS and axonal length were clearly shifted in time, with the AIS slowing down exchanges at earlier time points than axonal length, providing a compelling illustration of time-dependent filtering. It will be interesting for future studies to model these dynamics in naturally occurring neuronal morphologies. The approach described here provides and experimental and conceptual roadmap to do so.

## Discussion

Here we describe a versatile and unbiased photobleaching approach to study protein compartmentalization in developing neurons. With this assay, we found that while small cytoplasmic proteins ultimately pass through the AIS and reach comparable levels in these two compartments when expressed over hours, the AIS markedly reduces the exchanges of these proteins over minutes. Thus, small cytoplasmic proteins are compartmentalized by the AIS in a time-dependent manner.

FRAP studies typically focus on defined regions that are chosen before photobleaching. When applied to discontinuous structures, including organelles or membrane domains such as the AIS, reproducible positioning of these regions require the use of fiducial markers, for example a fluorescently-tagged structural protein of the AIS (Dumitrescu et al., 2016). This in turn reduces the number of fluorescence channels that are available for the experiment and subsequent retrospective labeling. Here we show that whole-cell photobleaching can be performed with multiple probes and in multiple cells in parallel, and reveals the location of protein filtering at the AIS without prior knowledge of its position. Further, we provide proof of principle that this assay can readily be combined with retrospective immunolabeling and super-resolution imaging, thus paving the way for future correlative live-cell/high-resolution structural imaging studies.

Super-resolution fluorescence microscopy has enabled important progress in our understanding of the ultrastructure of the neuronal F-actin cytoskeleton and its impact on membrane dynamics and intracellular signaling in axons and dendrites (Albrecht et al., 2016; van Bommel et al., 2019; Zhou et al., 2019). The present results suggest that periodic βII-spectrin ring spacers may also influence the diffusion of cytoplasmic proteins throughout the entire neuron, maybe by facilitating the formation of a cytoplasmic F-actin mesh and stable anchoring points in specific segments of neuronal processes.

More generally speaking, by preventing surface membrane proteins and specific intracellular membrane cargo from entering the axon (Winckler et al., 1999; Nakada et al., 2003; Song et al., 2009), the AIS ensures that distinct proteins can be targeted and restricted to the somatodendritic compartment or the axon. This compartmentalization is thus implicitly seen as a binary feature (e.g., excluded vs. allowed in the axon). In contrast, our results indicate that small cytoplasmic proteins – which are not intrinsically excluded from the axon – may be effectively compartmentalized in the somatodendritic compartment over specific time intervals (here tens of minutes).

Our data show that the diffusion of surface transmembrane proteins is slow enough that a large fraction of the somatic pool of these proteins can be targeted without affecting their dendritic or axonal pools, and so without actual diffusion barriers. The ability of the AIS to fence these proteins out of the axon (Winckler et al., 1999; Nakada et al., 2003; Albrecht et al., 2016) may thus be essential only to maintain the effective compartmentalization of these proteins over more extended time scales (many hours to days). In stark contrast, the diffusion of cytoplasmic proteins is so fast that the compartmentalization of these proteins requires physical barriers such as the AIS, and is only effective over relatively short time periods, typically a few minutes.

In the present case, fluorescence loss in the axon implies exchange of both unbleached and bleached molecules between the soma and the axon. Filtering at the AIS thus most likely impacts proteins entering the axon and proteins exiting the axons. However, based on this assay, we cannot formally rule out that this filtering shows some directionality. Indeed, as shown for the protein Tau, phospho-dependent binding to microtubules enables asymmetric filtering at the AIS, allowing anterograde flow but preventing retrograde movements (Li et al., 2011). Local reduction of protein diffusion and direct interaction with the cytoskeleton at the AIS thus likely enable elaborate filtering mechanisms to dynamically control the compartmentalization of specific pools of cytoplasmic proteins.

With half-lives ranging from hours to days (Cohen et al., 2013; Hanus and Schuman, 2013), protein stability varies greatly. Yet, the lifetime of the various possible posttranslational modifications of the same proteins ranges from seconds to many days and is considerably more variable (Kennedy et al., 2004; Nørregaard Jensen, 2004). It is thus conceivable that although a cytoplasmic protein may live long enough to pass the AIS, a more labile pool of the same protein – say its phosphorylated form – may be effectively excluded from the axon. The filtering properties of the AIS may thus be set so as to ensure that signaling via ubiquitous factors that are distributed throughout the entire cell can be done in a compartment specific manner. The present study thus opens new experimental and conceptual avenues to understand how the AIS controls neuronal compartmentalization over specific time scales. For example, real-time imaging of PKA activity with cAMP reporters showed that global PKA activation with forskolin induces a strong and local axonal response in DIV5 neurons but fails to do so in DIV3 neurons (Gorshkov et al., 2017). This compartmentalization of PKA signaling thus coincides with protein filtering at the AIS and seems to occur without obvious changes in the distribution of PKA and it anchoring proteins throughout the somadendritic and axonal compartments. Underlying molecular mechanisms are still not deciphered but are likely linked to the filtering properties of the AIS.

## Materials and Methods

### Primary Neuronal Culture

Sprague-Dawley (Charles River Laboratories) male and female rat pups of postnatal day 0 or 1 were used to prepare dissociated primary hippocampal neurons as previously described (Hanus et al., 2016). Neurons were routinely plated at a density of 40 × 10^3^ cells per cm^2^ on glass-bottom culture dishes (MatTek Corp., Hanus et al., 2016) coated with Poly-D-lysine (Millipore).

### Plasmids and Transfection

The mCherry, tdTomato, EGFP, and pHluo-TM plasmids were described previously (Cui-Wang et al., 2012). Neurons were transfected with Lipofectamine 2000 and Combimag (Life Technologies, OZ Biosciences) according to the manufacturers instructions.

### Live Cell Imaging and Photobleaching

DIV2-6 neurons were cotransfected with pHluoTM and mCherry (Figures 1, 3–5, 7) or EGFP and tdTomato (Figures 6, 8) as described above and imaged 24 h post-transfection. Neurons were then washed and monitored at 37°C in standard E4 medium (150 mM NaCl, 3 mM KCl, 15 mM glucose, 10 mM HEPES, pH 7.4) supplemented with 2 mM CaCl_2_, 2 mM MgCl_2_. Confocal imaging was performed using 20x objectives (NA = 0.8) on Zeiss Observer Z1 or Leica DMi8 inverted microscopes equipped with CSUX1 spinning disk units (Yokugawa, Inc.), high-precision motorized x,y,z stages (World Precision Instrument), EM-CCD (CoolSnap, Photometrics) or CMOS (Hamamatsu) cameras, and custom diode-laser illumination modules (3I Intelligent Imaging Innovations, Inc., Erol, SA). Neurons were photobleached in defined regions of interest (see below) by local irradiation (∼150–250 ms per round) with 488 nm or 561 lasers (50 and 100 mW, respectively) coupled to high speed x,y scanners (Vector, IMAGXCELL, SA).

FLIP experiments were performed in 3–5 cells in parallel. At each x,y position (i.e., each cell), z-stacks of GFP and mCherry channels (7–8 planes) were acquired with 0.75–1.25 μm z-steps. Photobleaching was combined with picture acquisition and was done in 7.5 μm-wide disks positioned on the soma, in the middle z-plan at each time point, starting after the 2nd or the 4th time point. Total acquisition/photobleaching time was ∼1.2 s (i.e., each z-stack) per channel, for a total of ∼2.5 s per cell for each time-point.

### Image Analysis

Pictures were processed and quantified using ImageJ or Metamorph (Molecular Devices). All experiments were conducted by imaging multiple locations in parallel, causing a small XY drift in most time-lapse sequences, thus requiring *post hoc* image alignment. Z-maximum intensity projections were thus aligned using the Image Stablizer plugin^1^, based on the pHluoTM channel (or otherwise unbleached GFP) because of the much slower fluorescence decay of this probe compared to mCherry.

Fluorescence levels were measured after subtraction of background fluorescence outside the cells.

Fluorescent decay was analyzed pixel-by-pixel using Pixbleach (Wüstner et al., 2012) and fitted with a mono-exponential function in the form :

$$I\,\left(t\right)={\text{I}}_{0}\times {\text{e}}^{\left(\frac{-\text{t}}{\tau }\right)}+{\text{I}}_{f}$$

With I, fluorescence at time t; I_0_ the initial fluorescence, I_f_ the fluorescence plateau, and τ (tau) the decay time constant. In brief, pixels of interest were selected by setting a minimal decay threshold and temporal (0.5–1.5) and spatial Gaussian filters (0.5–3) applied to the sequences to decrease noise. Fluorescence decay at each pixel was then fitted at each pixel with up to 30 iterations and estimated decay parameters (i.e., I_0_, τ) color-coded to generate heat maps of fluorescence decay throughout the cell.

As shown by analysis in 2 × 2 pixel-regions of interest and whole-cell heat-maps of RMSE values (Supplementary Figure S1), fluorescent decay in neurites was overall well fitted with mono-exponential functions (*R*^2^ = 0.97–0.99). Tau values calculated in the present study thus provide good estimates of protein diffusion throughout neurons but, as shown by trends in the variation of RMSE values during photobleaching (Supplementary Figures S1D,H), were affected by small systematic bias. Probably due to protein filtering by nuclear pores, goodness of fit was lower in the soma (Supplementary Figures S1B,F). In some neurons, the depletion of freely moving molecules revealed the presence of insoluble aggregates whose dynamics indicated intermittent active transport along microtubules, locally lowering the goodness-of-fit of fluorescent decay. These locations were clearly visible on RMSE heat maps (see Supplementary Figure S1B) and were excluded in analyses of Tau distribution along axons.

The proportion of neurons exhibiting filtering at the AIS was calculated as the fraction of neurons where Tau values at 60 μm in the axon were at least 1.5-fold higher than at 5 μm.

### Immunocytochemistry and Antibodies

Neurons were immunolabeled essentially as described previously (Hanus et al., 2016). In brief, neurons were rinsed in PBS, fixed for 15 min in 4% PFA, blocked and permeabilized for 30 min in a blocking buffer consisting of 0.15% Triton X100, 10% normal goat serum (Life Technologies) and 2% fish skin gelatin (Sigma) in PBS, and then incubated with primary and secondary antibodies in diluted (2-fold) blocking buffer. The following antibodies were used at the indicated dilutions: mouse anti-AnkG (UC Davis/NIH NeuroMab Facility, ref. 75-146; 1:2000), guinea pig anti − MAP2 (Synaptic Systems, ref. 188 004; 1:3000), mouse anti-βII-spectrin (BD Transduction Laboratories, ref 612562; 1:200), donkey anti-guinea pig – DyLight 405 (Jackson Labs, ref. 706-475-148; 1:800), goat-anti-mouse-AlexaFluorA647 (Life Technologies, ref. A21236; 1:750).

For retrospective confocal imaging after immuno-cytochemistry, cells were imaged using a 20x 0.8 NA Plan-Apochromat objective on a Zeiss LSM780 laser point scanning confocal microscopes. Maximum projections of the resulting z-stacks were then aligned to decay heat maps in ImageJ after adjustment of pixel sizes in the two sets of images. STORM images were acquired on a N-STORM microscope (Nikon Instruments), outfitted with 405 nm and 647 nm solid-state lasers, a 100x NA 1.49 objective and an Ixon DU-897 camera. Imaging was performed as described in Leterrier et al. (2015). A cylindrical astigmatism lens was placed in the optical path to acquire 3D information on fluorophore localization (Huang et al., 2008). 3D-coordinates of single fluorochromes and construction of super-resolution images were extracted and performed with the ThunderSTORM ImageJ plugin (Ovesný et al., 2014). Autocorrelation analyses were performed with the Stats^∗^ package of RStudio (R version 3.5.3).

### Computational Modeling

mCherry FLIP experiments in simplified neuron geometries were simulated using a three-dimensional diffusion-reaction model implemented in the Virtual Cell Modeling and Simulation Framework (Cowan et al., 2012; Li et al., 2015). Neuron geometries were defined according to the morphology of cultured neurons, and consisted of a spherical cell body of 20 μm diameter, a cylindrical dendrite with a diameter of 2 μm and length of 100 μm, and a cylindrical axon with a diameter of 1 or 2 μm and a length of 200 or 300 μm. We first set up minimal conditions in which there were only three molecular species: a fluorescent mCherry, a photobleached mCherry (both diffusible) and a non–diffusible “laser” catalyzer triggering the irreversible conversion of fluorescent into photobleached mCherry. Reactions were modeled as first order kinetics. The model was simulated in a mesh of 0.5 μm^3^ voxels 10 μM of diffusible fluorescent mCherry (diffusion coefficient: 20 μm^2^/s) was equally distributed in the neuron. For simulating FLIP experiments, the catalyzer (“laser”) was activated in the soma every 30 s. Simulation results were quantified in the same way as the experiments. For simulation of the AIS, two other species were added to the model: a slow diffusible fluorescent mCherry (5 μm^2^/s) and a non-diffusible AIS catalyzer spatially restricted between 10 and 30 μm in the proximal segment of the axon. This chemical reaction mimicked the diffusion barrier of the AIS that locally slows down the diffusion of soluble molecule. The core of the model can be found in the Virtual Cell public database via the VCell software.

### Statistics

Curve fitting and statistical analyses were performed in Prism (GraphPad). Data are presented as means ± SEM. The number of neurons used for quantification are indicated in the figure legends. Experiments were done in the indicated number of neurons, which were taken from 4 to 8 individual cultures dishes from 2 to 4 neuronal cultures. Multiple comparisons (N > 2 groups) were assessed with Kruskal Wallis’ and Dunn’s multicomparison tests. Comparisons of matched pairs were assessed with Wilcoxon’s test. Comparisons of fractions were assessed with Fisher’s exact test.

## Data Availability Statement

The datasets generated for this study are available on request to the corresponding author.

## Ethics Statement

All the experiments involving animals (i.e., postmortem tissue removal) that were performed for this publication were carried out in accordance with the European directive 2010/63/EU, the guidelines of the Federation of Laboratory Animal Science Associations (FELASA) and were reviewed and approved by the Comité d’Ethique en Matière d’Expérimentation Animale Paris Descartes (CNREEA #34, project #17-021).

## Author Contributions

LN, NG, TF, AL, DZ, and CH performed the experiments, analyzed the data, prepared the figures, and edited the manuscript. DM provided the technical support. CH designed and supervised the project and wrote the manuscript. All authors contributed to the article and approved the submitted version.

## Conflict of Interest

The authors declare that the research was conducted in the absence of any commercial or financial relationships that could be construed as a potential conflict of interest. The handling editor declared a shared affiliation, though no other collaboration, with several of the authors TF, DM, DZ, and CH.

## Footnotes

1 https://www.cs.cmu.edu/~kangli/code/Image_Stabilizer.html

## Supplementary Material

FIGURE S1 Decay of mCherry fluorescence in FLIP experiments is well fitted by mono-exponential functions. **(A,B,E,F)** Shown are two examples of heat maps of mCherry decay Tau **(A,E)** and fit RMSE (Root Mean Square Error) heat maps **(B,E)**. Scale bars 50 μm. In **(B,F)**, note the higher RMSE values in the soma. **(C,G)** Plots of mCherry fluorescence decay over time in proximal (25 μm from soma) and distal axonal segments (100 μm from soma). Shown is fluorescence normalized to initial and plateau values (circles), mono-exponential fits (black lines), and corresponding values of Tau, *R*^2^, and RMSE. **(D,H)** Residuals of fitted plots shown in **(C,G)**.

MOVIE S1 FLIP of pHluoTM (pseudocolored, Movie S1) and mCherry (pseudocolored, Movie S2) in a DIV5 neuron. Time in min. The bleached zone (not shown) is located in the soma. In Movie S1, the depletion of pHluoTM remains located on the soma while dendrites retain their fluorescence.

MOVIE S2 FLIP of pHluoTM (pseudocolored, Movie S1) and mCherry (pseudocolored, Movie S2) in a DIV5 neuron. Time in min. The bleached zone (not shown) is located in the soma. In Movie S2, the fluorescence of mCherry disappears rapidly from the entire somatodendritic compartment. Scale bar 50 μm.

MOVIE S3 Individual Z-sections of the projection shown in Figure 6C.
