# Supplementary figures and images for: Whole-Cell Photobleaching Reveals Time-Dependent Compartmentalization of Soluble Proteins by the Axon Initial Segment
Source: Front Cell Neurosci. 2020 Jul 10;14:180. doi: 10.3389/fncel.2020.00180 (PMC7366827; doi:10.3389/fncel.2020.00180)

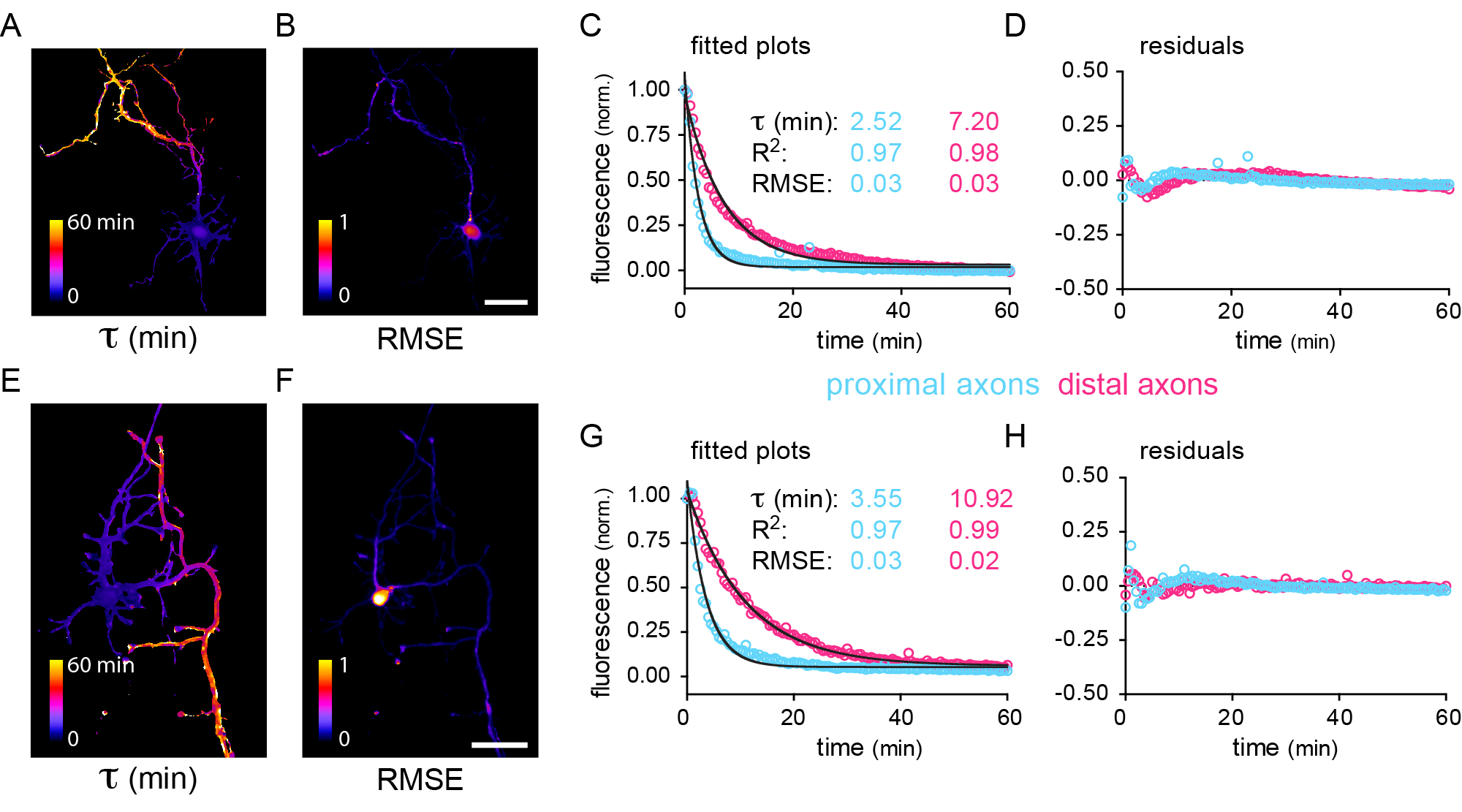

Supplement: FIGURE S1 — Decay of mCherry fluorescence in FLIP experiments is well fitted by mono-exponential functions. (A,B,E,F) Shown are two examples of heat maps of mCherry decay Tau (A,E) and fit RMSE (Root Mean Square Error) heat maps (B,E). Scale bars 50 μm. In (B,F), note the higher RMSE values in the soma. (C,G) Plots of mCherry fluorescence decay over time in proximal (25 μm from soma) and distal axonal segments (100 μm from soma). Shown is fluorescence normalized to initial and plateau values (circles), mono-exponential fits (black lines), and corresponding values of Tau, R2, and RMSE. (D,H) Residuals of fitted plots shown in (C,G). [file Image_1.jpg]
